# Indirect comparison between immune checkpoint inhibitors and targeted therapies for the treatment of melanoma

**DOI:** 10.7150/jca.32638

**Published:** 2019-10-15

**Authors:** Minliang Wu, Yuchong Wang, Yalong Xu, Ji Zhu, Chuan Lv, Mengyan Sun, Rui Guo, Yu Xia, Wei Zhang, Chunyu Xue

**Affiliations:** 1 Department of Plastic Surgery, Changhai Hospital, Second Military Medical University, Shanghai 200433, China; 2 Department of Urology, Changhai Hospital, Second Military Medical University, Shanghai 200433, China

**Keywords:** melanoma, indirect comparison, targeted therapy, immune checkpoint inhibitor

## Abstract

**Background:** This systematic review and meta-analysis aims to provide comparative and quantitative data about immune checkpoint inhibitor (IMM) and targeted therapy (TAR) in this work.

**Methods:** A literature search was performed with PubMed, Embase, PMC database, and Web of Science databases to identify relevant studies. Hazard ratios (HRs) for overall survival (OS) and progression-free survival (PFS), and odds ratios (ORs) for overall response rate (ORR) were estimated.

**Results:** Eighteen manuscripts were ultimately utilized for indirect comparisons. In general, both TAR and IMM can prolong the PFS either by monotherapy, combination therapy with chemotherapy or adjuvant therapy. BRAF inhibitor monotherapy showed superiority over anti-CTLA-4 in OS (HR: 1.28, 95%CI: 0.93-1.75) and best ORR (OR: 12.57, 95%CI: 6.63-23.82), as well as longer PFS (HR: 1.63, 95%CI: 1.00-2.67) and higher best ORR (OR: 3.29, 95%CI: 1.94-5.55) compared with anti-PD-1. However, MEK inhibitor monotherapy showed no priority. When combined with chemotherapy, anti-CTLA-4 showed marginally advantages over MEK inhibitor in OS (HR: 0.68, 95%CI: 0.44-1.03), however no advantage in PFS (HR: 1.12, 95%CI: 0.76-1.64), or ORR (OR: 1.78, 95%CI: 0.70-4.49). For post-operational melanoma patient, adjuvant TAR and adjuvant IMM showed no difference in OS (HR: 1.14, 95%CI: 0.82-1.58) or PFS (HR: 1.20, 95%CI: 0.79-1.83). Moreover, the high-rate adverse events and underlying diseases should be considered during the application of those agents.

**Conclusions:** For the unresectable late-stage melanoma, IMM may be a better choice for the combined treatment with chemotherapy. If the chemotherapy is not tolerable for patients, BRAFi involved TAR can be considered.

## Introduction

Cutaneous melanoma is an aggressive and deadly form of skin cancer. Globally, approximately 350,000 melanoma occurred every year and was responsible for 1,600,000 disability-adjusted life year each year.^1^ Late stage and metastatic melanoma is not candidate for surgical resection, systematic chemotherapy (CHE) should be applied routinely to eradicate unresectable and metastasized tumors. Besides chemotherapy, biological therapy, skin-directed therapy and radiation therapy are other widely used adjuvant therapies in melanoma treatment. However, these treatments have limited efficacy due to poor tissue selectivity, high toxicity, and strong drug resistance.

The development of immune checkpoint inhibitor (IMM) has changed the therapeutic selection of melanoma. Anti-CTLA-4 agents and anti-PD-1 agents are two kinds of IMMs recommended for patients with metastatic or unresectable disease.^2^ Ipilimumab, an anti-CTLA-4 agent, showed a statistically significant improvement in overall survival (OS) in patients with advanced melanoma^3^. Accordingly, anti-PD-1 treatment demonstrated ideal efficacy through increase T-cell antitumor activity even with patients resist to ipilimumab.^4^,^5^ Moreover, the anti-CTLA-4/anti-PD-1 combination therapy significantly improved response and progression-free survival (PFS) compared with monotherapy in unresectable stage III or stage IV disease, however, with increased toxicity 6-8

Another effective therapy is targeted therapy (TAR), the current recommended agents in this category include selective BRAF inhibitor (BRAFi), MEK inhibitor (MEKi) and KIT inhibitor (KITi).^9^ Based on the fact that approximately 50% of melanoma harbor BRAF gene activating point mutations^10^, development and approval of BRAFi have been applied in melanoma patients. Compared with chemotherapy, BRAFi monotherapy demonstrates efficacy in response rate, PFS, and OS for patients with previously untreated stage IV or unresectable stage III melanoma.^11^,^12^ Furthermore, the combination of BRAFi and MEKi has better efficacy than monotherapy.^13^-^15^ For patients with BRAF mutations, selection between first-line checkpoint immunotherapy and BRAFi can be difficult given the lack of comparative phase III clinical trials.

The current published data have only compared TARs or IMMs vs. chemotherapy or placebo. Meta-analysis is also limited to be conducted to compare monotherapy with combination therapy of the same type agents^13^-^16^, and IMMs or TARs with traditional chemotherapy^3^-^5^,^17^,^18^. No head-to-head RCT designed the direct comparison between those two kinds of treatments. The network meta-analysis provides a promising method to compare those treatments which have not been directly compared in RCT but being compared to a common comparator. We aim to provide a reference for physicians' decision making in the process of melanoma treatment.

## Materials and methods

### Literature search and article selection

A literature search was performed of the PubMed, Embase, PMC database, Web of Science databases and clinicaltrials.gov using following algorithm: (immune checkpoint inhibitor OR targeted therapy) AND melanoma AND clinical trial, and the algorithm (vemurafenib OR PLX4032 OR dabrafenib OR GSK2118436 OR LGX818 OR trametinib OR GSK-1120212 OR cobimetinib OR GDC-0973 OR ipilimumab OR MDX-010 OR tremelimumab OR CP-675,206 OR nivolumab OR MDX-1106 OR pembrolizumab OR MK-3475) AND melanoma AND clinical trial was also been used. All papers were available in full text and the criteria were confined to original articles conducted with human species and published in English. Two reviewers (MLW and YCW) independently screened titles and abstracts in duplicate, all conflicts were resolved by consensus or with a third reviewer (YLX).

### Inclusion and exclusion criteria

The following inclusion and exclusion criteria were used: (1) the phase II or III randomized controlled trials (RCTs) with IMM or TAR agents alone or combined with chemotherapy compared to chemotherapy or placebo; (2) the study reported on at least one of the following outcomes: OS, PFS, overall response rate (ORR), and/or adverse events (AEs); (3) if multiple publications of the same trial were retrieved, the most recent publication was utilized; (4) articles with incomplete literature data were excluded.

### Evaluation of study quality and data collection

The Oxford Centre for Evidence-Based Medicine criteria were used to estimate the levels of evidence. The methodological quality assessment of the RCTs was conducted independently by MLW and YLX using the Jadad Scale. The data were extracted by JZ and CL using predefined data collection forms and the extracted data were verified independently by MYS.

### Statistical analysis

The analyzed endpoints for the study included OS, PFS and best ORR. For PFS and OS, we extracted the hazard ratio (HR) and confidence interval (CI) when available; while for best ORR, we extracted the odds ratio (OR) and CI. We used the Cochran *Q* statistic to estimate statistical heterogeneity and the *I^2^* statistic to quantify inconsistency: homogeneity was rejected when the *Q* statistic *P* < 0.10 or the *I^2^* > 50%. A fixed-effect model was used to estimate the weighted median values (or combined rates) and the 95% CIs if there was no evidence of heterogeneity; otherwise, a random-effect model was used. ITC version 1.0 software (Canadian Agency for Drugs and Technologies in Health, Ottawa, Ontario, Canada) and Stata version 12.0 software (StataCorp, College Station, TX, USA) were utilized for the analysis.

## Results

### Study characteristics

A total of 366 articles were initially retrieved in our study, 141 records were removed due to duplication, 205 were deemed ineligible after title and abstract screening, leaving 20 studies for full-text review (Supplementary Figure 1). Sixteen RCTs were ultimately included for indirect comparisons between IMM and TAR as the treatment of melanoma, including 12 phase III RCTs^7^,^17^-^29^ and 4 phase II RCTs^30^-^33^. However, because there were two trials involving two articles respectively for the absences of some endpoints in a single article, the number of included manuscripts was 18. The methodological quality of the included RCTs was high for all the trials (Jadad Scale: 4-5 of 5 points). We divided those final 16 trials into three subgroups: group 1, comparison between IMM (or TAR) and chemotherapy; group 2, comparison between IMM (or TAR) combined with chemotherapy and chemotherapy alone; group 3, comparison between adjuvant IMM (or TAR) and placebo. In detail, group 1 was further divided into anti-CTLA-4 vs. CHE, anti-PD-1 vs. CHE, BRAFi vs. CHE, MEKi vs. CHE; group 2 was further divided into anti-CTLA-4+CHE vs. CHE, MEKi+CHE vs. CHE. The characteristics of these trials are summarized in Supplementary Table S1.

### PFS

**Figure 1:**
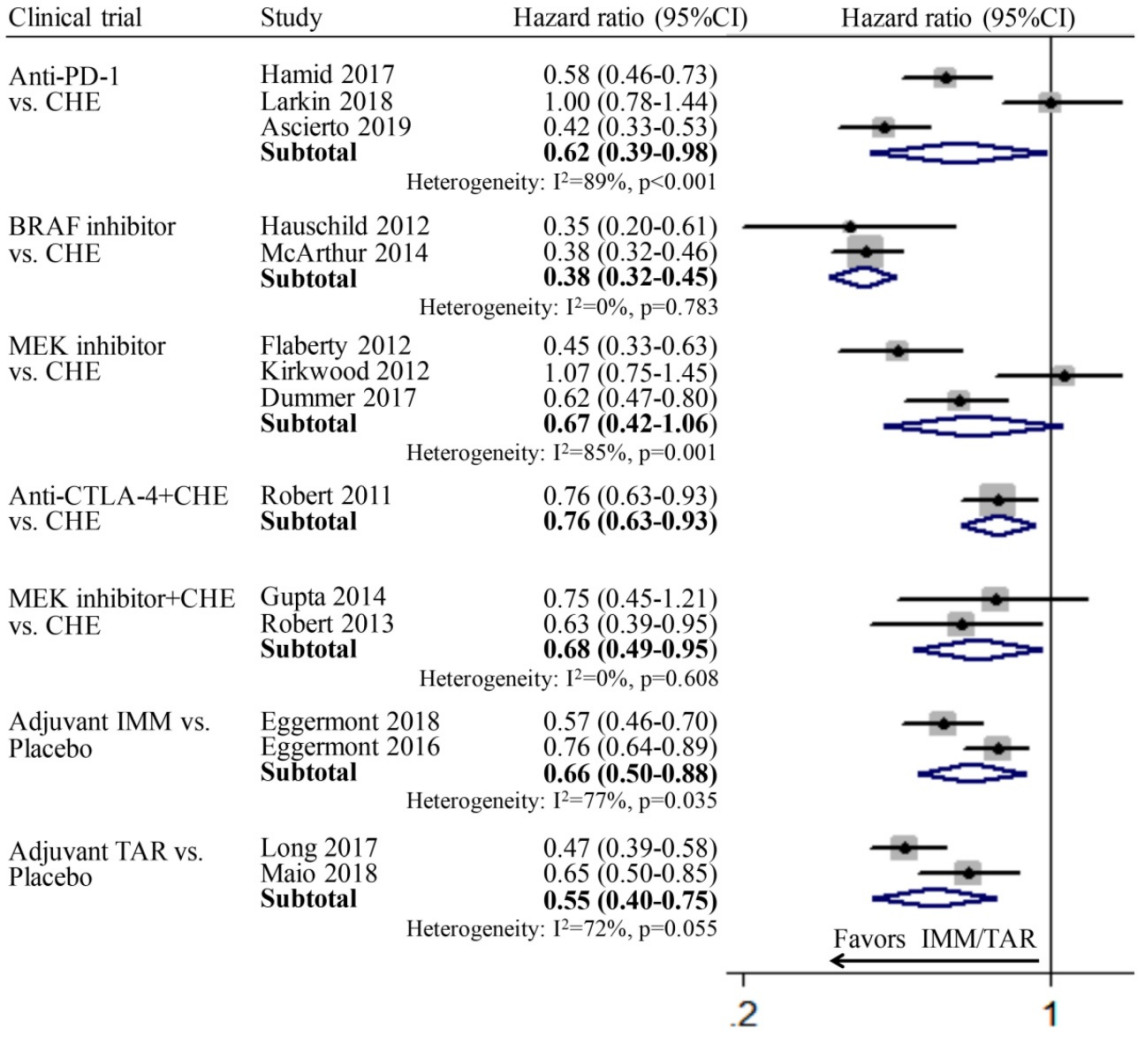
Individual study and pooled HR estimates of progression-free survival between targeted therapy and immune therapy.

The pooled respective HRs for anti-PD-1 vs. CHE, BRAFi vs. CHE, MEKi vs. CHE, anti-CTLA-4+CHE vs. CHE, MEKi+CHE vs. CHE, adjuvant IMM vs. placebo, and adjuvant TAR vs. placebo all showed statistically significant difference. For subgroup MEKi vs. CHE, the pooled HR is 0.67 (95%CI: 0.42-1.06), which showed not significant but relative difference. It indicated the efficacy of those three various therapeutic modes involved IMM or TAR are better than chemotherapy or placebo. The absence of pooled PFS for subgroup anti-CTLA-4 vs. CHE was due to the lack of relevant data in the included study (Figure 1).

### OS

**Figure 2:**
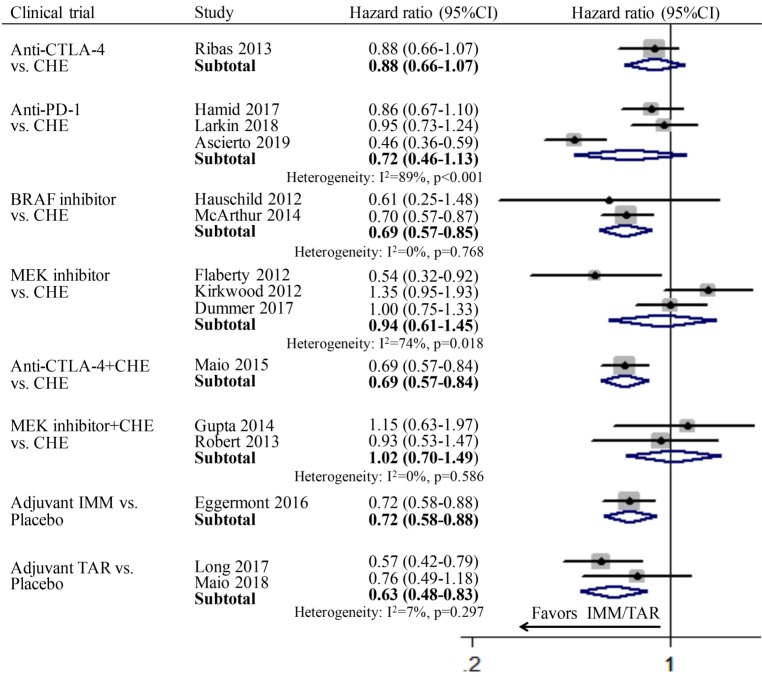
Individual study and pooled HR estimates of overall survival between IMM and TAR.

Since only one study was included in subgroup anti-CTLA-4 vs. CHE, anti-CTLA-4+CHE vs. CHE, adjuvant IMM vs. placebo respectively, thus the pooled OS was calculated directly using the data in the published literatures. In the group of monotherapy, anti-CTLA-4 (HR: 0.88; 95%CI: 0.66-1.07), anti-PD-1 (HR: 0.72; 95%CI: 0.46-1.13), and MEKi (HR: 0.94; 95%CI: 0.61-1.45) showed no improvement of OS compared to chemotherapy; while only BRAFi (HR: 69; 95%CI: 0.57-0.85) achieved significant longer OS than chemotherapy. In the group of combination therapy, anti-CTLA-4 combined with chemotherapy showed significant advantage in OS compared with chemotherapy alone (HR: 0.69; 95%CI: 0.57-0.84), whereas the combination of MEKi and chemotherapy showed no superiority (HR: 1.02; 95%CI: 0.70-1.49). In the subgroup of adjuvant therapy, both IMM (HR: 0.72; 95%CI: 0.58-0.88) and TAR (HR: 0.63; 95%CI: 0.48-0.83) demonstrated significantly better OS than placebo (Figure 2).

### ORR

**Figure 3:**
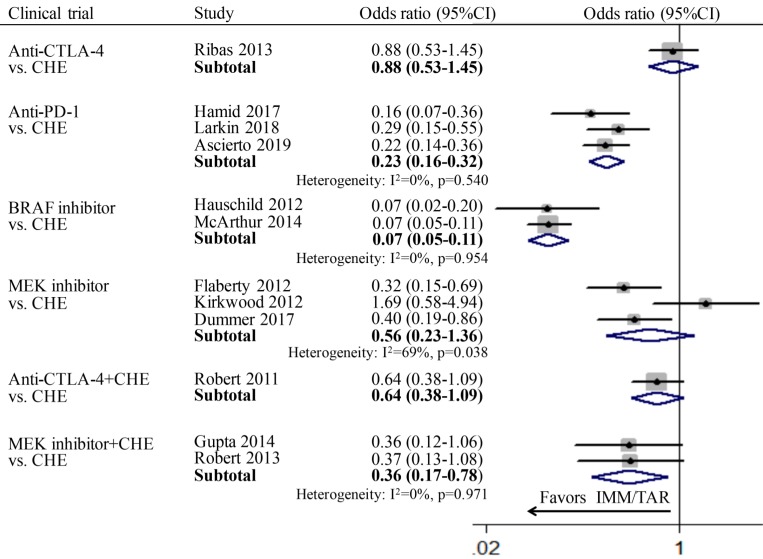
Individual study and pooled OR estimates of overall response rate between targeted therapy and immune therapy.

For the comparison between TAR (or IMM) monotherapy and chemotherapy, anti-PD-1 (OR: 0.23; 95%CI: 0.16-0.32) and BRAFi (OR: 0.07; 95%CI: 0.05-0.11) achieved higher ORR than chemotherapy. However, for subgroups of anti-CTLA-4 vs. chemotherapy (OR: 0.88, 95%CI: 0.53-1.45) and MEKi vs. chemotherapy (OR: 0.56, 95%CI: 0.23-1.36), no significant difference was found. In addition, the combination therapy of MEKi and chemotherapy (OR: 0.36; 95%CI: 0.17-0.78) increased the ORR compared to chemotherapy alone, whereas no improvement of ORR was observed for anti-CTLA-4 combined with chemotherapy (OR: 0.64; 95%CI: 0.38-1.09) (Figure 3).

### Indirect comparison outcomes

**Figure 4:**
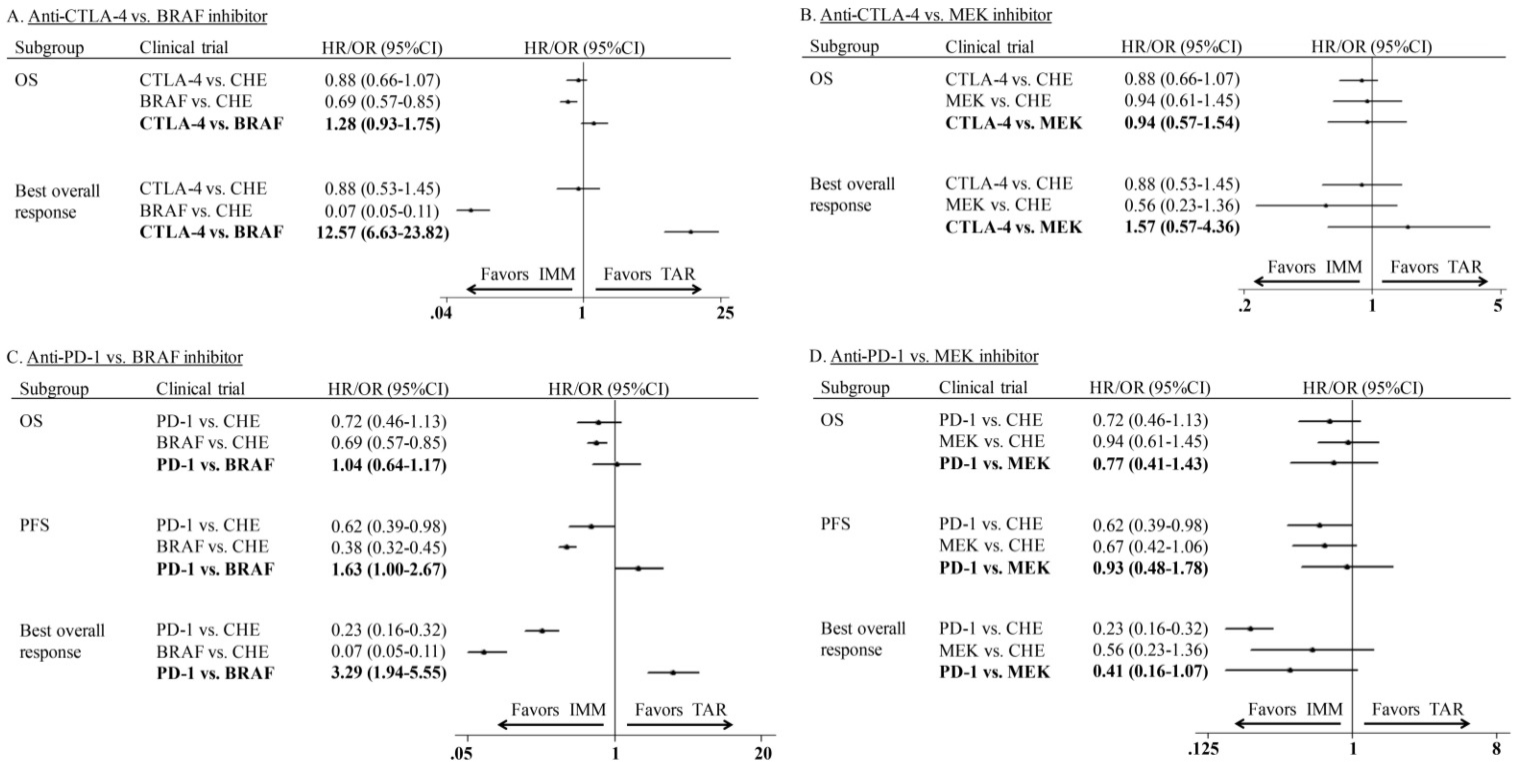
Indirect comparison of progression-free survival, overall survival and overall response rate between IMM and TAR monotherapy.

Indirect comparisons of monotherapy were conducted between IMM (anti-CTLA-4 and anti-PD-1) and TAR (BRAFi and MEKi). The indirect comparison of anti-CTLA-4 vs. BRAFi showed that BRAFi provided marginally longer OS (HR: 1.28; 95%CI: 0.93-1.75) and significantly higher ORR (OR: 12.57; 95%CI: 6.63-23.82) than anti-CTLA-4 agents (Figure 4A). However, no significant difference was shown between anti-CTLA-4 and MEKi in neither OS (HR: 0.94; 95%CI: 0.57-1.54) nor ORR (OR: 1.57; 95%CI: 0.57-4.36) (Figure 4B). The indirect estimate for anti-PD-1 vs. BRAFi showed the latter one had PFS (HR: 1.63; 95%CI: 1.00-2.67) and ORR (OR: 3.29; 95%CI: 1.95-5.55) advantage over the former one, whereas no difference was observed in OS (HR: 1.04; 95%CI: 0.64-1.17) of those two agents (Figure 4C). For subgroup of anti-PD-1 vs. MEKi, the close to significant difference was found between two agents in ORR (OR: 0.41; 95%CI: 0.16-1.07), but not OS (HR: 0.77; 95%CI: 0.41-1.43) or PFS (HR: 0.93; 95%CI: 0.48-1.78) (Figure 4D).

**Figure 5:**
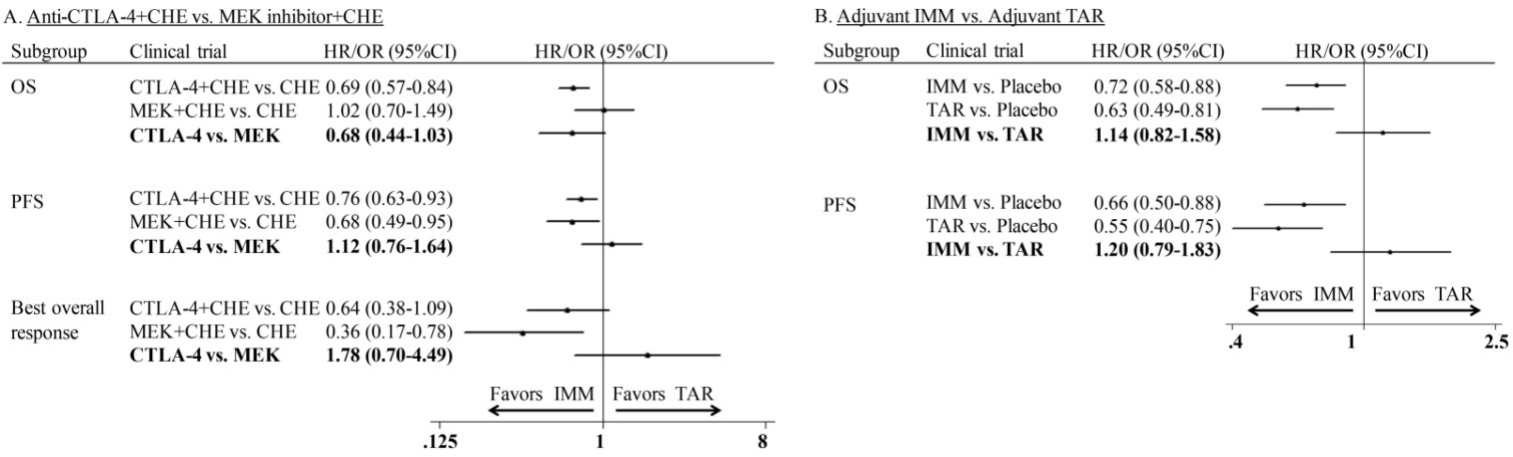
Indirect comparison of progression-free survival, overall survival and overall response rate of combination therapy and adjuvant therapy.

In the comparison of combination therapy, although no difference was found in PFS (HR: 1.12; 95%CI: 0.76-1.64) and ORR (OR: 1.78; 95%CI: 0.70-4.49), the combination of anti-CTLA-4 agent and chemotherapy showed superior OS than MEKi combined with chemotherapy (HR: 0.68; 95% CI: 0.44-1.03) (Figure 5A). In the comparison of adjuvant therapy, no advantage was found between IMM and TAR in either OS (HR: 1.14; 95%CI: 0.82-1.58) or PFS (HR: 1.20; 95%CI: 0.79-1.83) (Figure 5B).

### Adverse events

Generally speaking, the overall safety profile of IMM and TAR are tolerable, with manageable toxic effects appearing less frequently than chemotherapy. Diarrhea, pruritus, rash, fatigue, vomiting, peripheral oedema, and nausea are some of the most common toxic effects for patients treated with IMM or TAR. Compared with IMM, skin-related toxic effects and secondary cutaneous lesions like hyperkeratosis, papillomas, palmar-plantar erythrodysaesthesia, dermatitis acneiform, cutaneous squamous-cell carcinoma and keratoacanthomas seemed to be more related to TAR. The ratio of grade 3-4 AEs are relatively less for IMM or IMM involved therapy than that for TAR (Table 1).

## Discussion

According to National Cancer Institute, there will be 92,000 estimated new cases of melanoma in 2018, taking up 5.3% of all new cancer cases. Moreover, it is estimated to cause 9,000 deaths in 2018, accounting for 1.5% of all cancer deaths. Traditionally, dacarbazine has been regarded as the first line treatment for melanoma since its approval in 1970s. Although the therapeutic options for melanoma have been developed, the survival prognoses are still poor and therapy decisions become more complicated than before^34^. This situation has been rapidly improved since the introduction of two new systemic therapies, IMMs and TARs^35^. However, there is yet no head-to-head phase Ⅲ clinical trial being conducted to compare TARs and IMMs for the treatment of melanoma.

A meta-analysis published in 2017 compared the impact of IMMs and TARs on efficacy and acceptability (the inverse of high grade toxicity) of melanoma patients.^36^ However, it had limitations of following two aspects: first, the efficacy analysis was restricted to PFS and detailed complications associated with those treatments were not provided; second, the therapeutic modes involved IMM or TAR including synergy with chemotherapy and adjuvant therapy were not analyzed and discussed either. In the present study, the various endpoints including OS, PFS and ORR have been indirectly compared between IMM or TAR involved therapies, and the detailed complications of these two agents have been systemically illustrated and analyzed. Furthermore, not only the monotherapy mode but also the combination and adjuvant therapies are studied. The newly published studies in recent two years are updated and included into our network meta-analysis. Our study aims to comprehensively compare the efficacy and safety of IMM or TAR involved therapeutic modes, and better inform the decision-making process of physicians.

The IMMs are a group of monoclonal antibodies that block co-inhibitory molecules such as CTLA-4 (expressed on activated CD4+ and CD8+ effector T-cells and regulatory T-cells), PD-1 (also expressed on activated effector T-cells) and its ligand PD-L1 (which is expressed on dendritic cells, activated T-cells, and tumor cells)^37^. As IMMs enjoy the superior efficacy compared to chemotherapy, which was confirmed by the pooled PFS and ORR results in our network meta-analysis, melanoma is the presently lead indication for the approval of checkpoint inhibitors^38^. The immunosuppressive action by anti-PD-1 works in the effector phase of the interaction between T lymphocytes and tumor cells, and the blockade of this agent seems to be more effective towards T-cell activation than CTLA-4 blockade. Maio *et al*. reported that ipilimumab, a first in class anti-CTLA-4 monoclonal antibody, resulted in long-term survival in approximately 20% of patients.^27^ Furthermore, two anti-PD-1 monoclonal antibodies, nivolumab and pembrolizumab, were showed to have greater efficacy than ipilimumab^4^,^5^. Our data also indicated that anti-PD-1 had a greater survival and response advantage than anti-CTLA-4 when compared to chemotherapy. However, there is still 60% of melanoma patients showing primary resistance to IMMs, and 20-30% of initial responders will develop acquired resistance at last^38^.

As the most frequent genetic alteration in melanoma, oncogenic mutations in BRAF gene are observed in 40-50% of patients, contributing to the constitutive activation of the MAPK pathway and oncogenic development^39^-^41^. Hence, the BRAFi such as vemurafenib and dabrafenib were developed to cure advanced melanoma by specifically targeting this driver mutation. Another therapeutic target is the signaling molecule MEK downstream of BRAF, with its blockade inactivating the MAPK pathway.^12^,^42^ Interestingly, BRAFi, not MEKi, was significantly superior to chemotherapy in pooled endpoints including PFS, OS and ORR according to our network meta-analysis results. Two major problems with TARs in BRAF-mutated melanoma are the occurrence of non-melanoma secondary skin cancer, and the development of resistance while on therapy.^24^ Studies showed BRAFi combined with MEKi could increase the medium PFS from 7-9 months with BRAFi monotherapy to 11-15 months.^13^,^14^,^42^ This might due to the MEKi can avoid reactivation of the MAPK pathway by BRAFi monotherapy, and thus reduce the skin toxicity.

The treatment goals for patients with advanced melanoma have two aspects: short-term alleviation and induction of durable remission, and how the current available therapeutic modes are used to optimize both remains unclear. IMMs and TARs each have substantial clinical benefits. Our indirect comparison between IMM and TAR showed the BRAFi monotherapy significantly improved the survival and response outcomes compared to both anti-CTLA-4 and anti-PD-1, which is consistent with the conclusion of a previous network meta-analysis about TAR and IMM^36^. It might be attributed to the following possible reasons. First of all, different from IMMs, TARs can kill tumor cell directly. A rapid response within days to a few weeks regardless of tumor burden and metastasis location is a typical feature of all BRAFi-based therapies^43^. Moreover, subgroup analyses from multiple BRAF trials have demonstrated particularly beneficial for the more advanced and aggressive melanoma, such as those with elevated LDH level or brain metastasis^44^. Whereas similar analyses for immunotherapies have tended to favor patients with less aggressive or advanced disease 44. At the same time, our indirect comparison also demonstrated the combination of anti-CTLA-4 and chemotherapy was superior to MEKi combined with chemotherapy in terms of OS. The additive or synergistic clinical activity achieved by combination of IMMs and chemotherapy might contribute to above phenomenon. Distinct chemotherapy agents may promote tumor immunity through a variety of mechanisms. Chemotherapy may render tumor cells more sensitive to T-cell-mediated immune attack by disrupting strategies that tumors use to evade immune recognition. Moreover, chemotherapy can enhance the strength of effector T-cell activity by upregulating co-stimulatory molecules or downregulating co-inhibitory molecules expressed on the tumor cell surface^45^,^46^. The analysis of the current available evidence in our study indicates when monotherapy is considered, TAR, especially BRAFi, should be put in priority; when combined with chemotherapy, IMM, especially anti-CTLA-4, should be considered firstly.

Observation is the standard of care after resection of melanoma in most countries^23^. However, recurrence of melanoma after definitive surgery is a substantial risk for patients with completely resected stage III melanoma. The adjuvant therapy with agents already approved or under clinical trials should be considered to prevent tumor relapse and metastasis, and ultimately improve survival outcomes. The agents previously approved for systemic adjuvant treatment of melanoma included dacarbazine, cisplatin, vinblastine, IL-2, interferon alfa-2b and pegylated interferon,^47^,^48^ which showed inconsistent improvements in OS along with substantial toxic effects^49^,^50^. In our subgroup analysis of adjuvant therapy, both IMMs and TARs demonstrated significantly better survival outcomes than placebo. However, indirect comparison did not discover statistical difference between IMM and TAR in terms of OS and PFS. It indicates that IMMs (for all melanoma) and TARs (for BRAF-mutant melanoma) will become the new standards of adjuvant therapy for resected stage III melanoma in the near future^38^.

Additional to the significant advantages IMM and TAR demonstrated in the adjuvant therapy for patients with high-relapse risk after surgical resection, their application in neoadjuvant therapy prior to surgery can reduce tumor burden, decrease local and distant recurrences. There have been abundant clinical trials to demonstrate this category of treatment (NCT02306850, NCT01972347, NCT02519322, NCT02303951, NCT02036086, etc). In the OpACIN trial, no surgery-associated AEs attributable to neoadjuvant ipilimumab plus nivolumab were observed, and the neoadjuvant pathologic response rate was up to 80%.^51^ Other clinical trials about neoadjuvant TAR reported shrunk unresectable stage III or oligometastatic stage IV melanoma tumors sufficiently to allow complete resection.^52^ The optimal dose and protocol need to be figured out to achieve ideal therapeutic effect for patients with high-risk melanoma.

The included studies reported a range of inflammatory side effects associated with IMMs, so-called immune-related AE (irAE). For anti-CTLA-4 agents, irAEs mainly affect the gastrointestinal system (diarrhea and colitis), skin (dermatitis and pruritus), liver (hepatitis and increased liver function tests), which are dose dependent.^53^ Differently, anti-PD-1-associated irAEs more often affect the lung (pneumonitis) and the thyroid gland (hyperthyroidism or hypothyroidism)^53^. Although the combination of nivolumab and ipilimumab achieved significant survival improvement compared with ipilimumab monotherapy in a phase III trial, the irreversible irAEs were reported more common and severely in combination therapy than those in monotherapy^54^. According to the included studies, BRAFi and MEKi presented different toxicity profiles between each other. The most frequently observed AEs in patients treated with BRAFi were arthralgia and fatigue, whereas diarrhea/colitis and rash were the most common ones among patients treated with MEKi. In addition, BRAFi and MEKi combination therapy had a good safety and tolerability profile. Common AEs comprised gastrointestinal symptoms including fatigue, nausea, diarrhea, vomiting, arthralgia, and palmoplantar skin reactions^23^. In clinical practice, the decision of therapeutic strategy should be made prudently and the patients who need combination therapy should be chosen cautiously.

We acknowledge that our study has some limitations. First, the eligible trials have generally consistent inclusion criteria, while some differences do exist, as shown in the Supplementary Table 1. In some trials testing TARs, all cases had BRAF-mutant melanoma, whereas some other trials investigating IMMs enrolled both wild-type and mutant BRAF melanoma. Second, several subgroup analyses had only one trial included such as anti-CTLA-4 vs. CHE, and anti-CTLA-4+CHE vs. CHE, which limited the evidence level of pooled data. Third, some of the evidence supporting survival priority was based on marginally significant advantage as the confidence interval cross the null value. This calls for further phase III RCTs directly comparing IMMs and TARs to provide evidence of high quality.

## Conclusion

In the absence of RCT directly comparing IMMs and TARs, our findings suggest that compared with chemotherapy, both IMMs and TARs, except MEKi, can significantly improve the survival or response outcomes for advanced melanoma by monotherapy. For the unresectable late-stage melanoma, IMM may be a better choice for the combined treatment with chemotherapy. If the chemotherapy is not tolerable for patients, BRAFi involved TAR can be considered. Either IMMs or TARs are recommended as the new standards of adjuvant therapy for resected stage III melanoma.

## Supplementary Material

Supplementary figures and tables.

## Figures and Tables

**Table 1:** Summary of adverse events of included studies

Clinical trial	Study	Adverse events, n (%)													
		All grades	Grade 3/4	Diarrhea/Colitis	Nausea	Fatigue	Pruritus	Rash	Vomiting	Decreased appetite	Pyrexia	Arthralgia	Peripheral oedema	Neutropenia	Constipation
IMM vs. CHE	Ribas 2013	312/325 (96)	170 (52)	166 (51)	109 (34)	106 (33)	100 (31)	106 (33)	74 (23)	67 (21)	53 (16)	NA	32 (10)	2 (0.6)	48 (15)
	Hamid 2017	125 (70)	24 (13)	18 (10)	11 (6.2)	44 (25)	39 (22)	23 (13)	4 (2.2)	11 (6.2)	NA	NA	NA	1 (0.6)	5 (2.8)
	Larkin 2018	266 (99)	126 (47)	49 (18)	33 (12)	86 (32)	59 (22)	38 (14)	9 (3.4)	18 (6.7)	14 (5.2)	22 (8.2)	NA	0 (0.0)	6 (2.2)
	Ascierto 2019	160 (78)	31 (15)	39 (19)	NA	NA	49 (24)	38 (18)	NA	NA	NA	NA	NA	NA	NA
TAR vs. CHE	Hauschild 2012	100 (53)	NA	NA	2 (1.1)	12 (6.4)	NA	NA	2 (1.1)	NA	20 (11)	10 (16)	NA	1 (0.5)	NA
	McArthur 2014	334 (99)	247 (73)	NA	128 (38)	156 (46)	85 (25)	138 (41)	72 (21)	73 (22)	71 (21)	189 (56)	68 (20)	2 (0.6)	NA
	Flaherty 2012	NA	NA	91 (43)	38 (18)	54 (26)	NA	121 (57)	27 (13)	NA	NA	NA	54 (26)	NA	30 (14)
	Kirkwood 2012	99 (100)	57 (58)	56 (57)	50 (51)	29 (29)	NA	NA	28 (28)	NA	16 (16)	NA	40 (40)	NA	12 (12)
	Dummer 2017	NA	NA	108 (40)	79 (29)	60 (22)	32 (12)	98 (36)	57 (21)	31 (12)	28 (10)	NA	NA	3 (1.1)	37 (14)
IMM+CHE vs. CHE	Robert 2011	244 (99)	139 (56)	90 (36)	NA	NA	73 (30)	61 (25)	NA	NA	91 (37)	NA	NA	NA	NA
TAR+CHE vs. CHE	Gupta 2014	NA	NA	32 (84)	19 (50)	28 (74)	NA	29 (76)	11 (29)	NA	NA	NA	15 (39)	NA	11 (29)
	Robert 2013	44 (100)	30 (68)	21 (48)	28 (64)	16 (36)	10 (23)	39 (89)	21 (48)	10 (23)	NA	NA	19 (43)	7 (16)	12 (27)
Adjuvant IMM vs. Placebo	Eggermont 2018	396 (78)	75 (15)	97 (19)	58 (11)	189 (37)	90 (18)	82 (16)	NA	NA	NA	61 (12)	NA	NA	NA
	Eggermont 2015	465 (99)	260 (55)	231 (49)	116 (25)	189 (40)	203 (43)	185 (39)	59 (13)	65 (14)	82 (17)	NA	NA	NA	NA
Adjuvant TAR vs. Placebo	Long 2017	422 (97)	180 (41)	144 (33)	172 (44)	204 (47)	NA	106 (24)	122 (28)	48 (11)	NA	120 (28)	58 (13)	NA	51 (12)
	Maio 2018	245 (99)	141 (57)	60 (24)	86 (35)	78 (32)	72 (29)	92 (37)	33 (13)	33 (13)	44 (18)	NA	NA	3 (1.2)	NA
