# Supplementary material for: Indirect comparison between immune checkpoint inhibitors and targeted therapies for the treatment of melanoma
Source: J Cancer. 2019 Oct 15;10(24):6114–23. doi: 10.7150/jca.32638 (PMC6856565; doi:10.7150/jca.32638)
Supplement: Supplementary file 1 — Supplementary figures and tables. [file jcav10p6114s1.pdf]

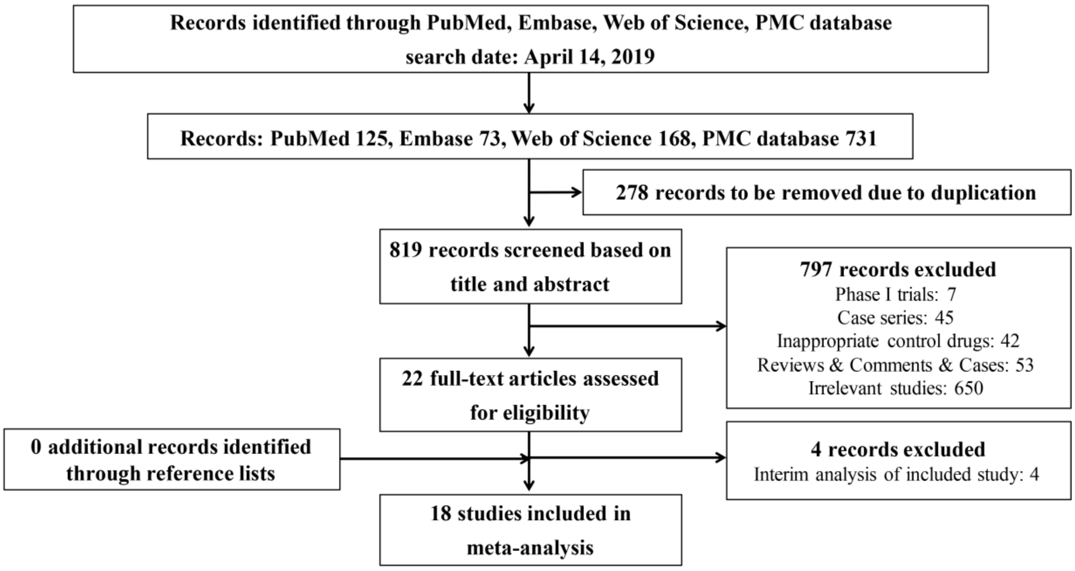

Supplementary Figure 1. PRISMA flow diagram of the search strategy.

Supplementary Table 1 Summary of comparative studies

Clinical trial	Study	Study design	LE	Study quality	Included patients	Cases, n			
						Total	Primary endpoint events	IMM/TAR arm	Control arm
IMM vs. CHE	**Ribas 2013**	RCT/Phase 3	1b	4	treatment-naive, unresectable stage IIIc or IV melanoma	655	534	328	327
	**Hamid 2017**	RCT/Phase 2	1b	5	untreated stage III (unresectable) or stage IV melanoma	540	466	180	179
	**Larkin 2018**	RCT/Phase 3	1b	5	unresectable,untreated stage III or IV melanoma without a BRAF mutation	405	244	272	133
	**Ascierto 2019**	RCT/Phase 3	1b	5	unresectable,untreated stage III or IV melanoma without a BRAF mutation	418	306	210	208
TAR vs. CHE	**Hauschild 2012**	RCT/Phase 3	1b	5	unresectable or metastatic melanoma and progressed after ipilimumab/ipilimumab+BRAF inhibitor	250	30	187	63
	**McArthur 2014**	RCT/Phase 3	1b	5	untreated, stage IV or unresectable stage III BRAF mutation-positive melanoma	675	399	337	338
	**Flaherty 2012**	RCT/Phase 3	1b	4	treatment-naive metastatic melanoma and whose tumour tissue was positive for BRAF mutations	322	64	214	108
	**Kirkwood 2012**	RCT/Phase 2	1b	5	unresectable stage III or IV melanoma	200	130	104	96
	**Dummer 2017**	RCT/Phase 3	1b	5	stage IIIC or IV melanoma harbouring an NRAS Gln61Arg, Gln61Lys, or Gln61Leu mutation	402	228	269	133
IMM+CHE vs. CHE	**Robert 2011/Maio 2015**	RCT/Phase 3	1b	5	unresectable stage IIIC or IV cutaneous melanoma with BRAF mutation	502	414	250	252
TAR+CHE vs. CHE	**Gupta 2014**	RCT/Phase 2	1b	5	patients with wild-type BRAF melanoma	83	54	41	42
	**Robert 2013**	RCT/Phase 2	1b	5	advanced BRAF-mutant cutaneous or unknown primary melanoma	91	66	45	46
Adjuvant IMM vs. Placebo	**Eggermont 2018**	RCT/Phase 3	1b	5	completely resected stage III melanoma	1019	351	514	505
	**Eggermont 2015/ Eggermont 2016**	RCT/Phase 3	1b	5	stage IIIA, IIIB or IIIC melanoma with no in-transit metastasis	951	376	475	476
Adjuvant TAR vs. Placebo	**Long 2017**	RCT/Phase 3	1b	5	completely resected, stage III melanoma with BRAF mutation	870	153	438	432
	**Maio 2018**	RCT/Phase 3	1b	5	histologically confirmed stage IIC–IIIA–IIIB or stage IIIC BRAF mutation-positive melanoma that was fully resected	498	82	250	248
